# Neck Proprioception Shapes Body Orientation and Perception of Motion

**DOI:** 10.3389/fnhum.2014.00895

**Published:** 2014-11-04

**Authors:** Vito Enrico Pettorossi, Marco Schieppati

**Affiliations:** ^1^Department of Experimental Medicine, University of Perugia, Perugia, Italy; ^2^Department of Public Health, Experimental and Forensic Medicine, University of Pavia, Pavia, Italy; ^3^Centro Studi Attività Motorie (CSAM), Fondazione Salvatore Maugeri (IRCSS), Scientific Institute of Pavia, Pavia, Italy

**Keywords:** self-motion perception, vestibular, proprioception, neck

## Abstract

This review article deals with some effects of neck muscle proprioception on human balance, gait trajectory, subjective straight-ahead (SSA), and self-motion perception. These effects are easily observed during neck muscle vibration, a strong stimulus for the spindle primary afferent fibers. We first remind the early findings on human balance, gait trajectory, SSA, induced by limb, and neck muscle vibration. Then, more recent findings on self-motion perception of vestibular origin are described. The use of a vestibular asymmetric yaw-rotation stimulus for emphasizing the proprioceptive modulation of motion perception from the neck is mentioned. In addition, an attempt has been made to conjointly discuss the effects of unilateral neck proprioception on motion perception, SSA, and walking trajectory. Neck vibration also induces persistent aftereffects on the SSA and on self-motion perception of vestibular origin. These perceptive effects depend on intensity, duration, side of the conditioning vibratory stimulation, and on muscle status. These effects can be maintained for hours when prolonged high-frequency vibration is superimposed on muscle contraction. Overall, this brief outline emphasizes the contribution of neck muscle inflow to the construction and fine-tuning of perception of body orientation and motion. Furthermore, it indicates that tonic neck-proprioceptive input may induce persistent influences on the subject’s mental representation of space. These plastic changes might adapt motion sensitiveness to lasting or permanent head positional or motor changes.

## Introduction

To many aged professors of physiology (and young students as well), the term proprioception promptly calls to mind the tendon-tap reflex, i.e., the “monosynaptic” reflex elicited by a tap onto the patellar or Achilles’ tendon and the consequent leg extension and foot plantarflexion, respectively. This phenomenon has been given such straightforward explanation (Ia spindle afferent fibers – motoneurons – homonymous muscle contraction) that no-one would have thought to doubt on the vital role of proprioceptive reflexes in all aspects of human movement, and in particular in the control of quiet upright stance or gait. Some doubts, however, should emerge from the simple observation that people devoid of deep muscle reflexes, e.g., patients with Holmes-Adie’s syndrome (Adie, 1932) or Charcot-Marie-Tooth type Ia (Nardone et al., 2000), can stand up and walk (Mazzaro et al., 2005) almost as well as if their monosynaptic reflexes were present and brisk. Still more reservations should arise from observing the decreased excitability of the Achilles’ reflex during stance (Bove et al., 2006a) and locomotion (Crenna and Frigo, 1987) compared to laying down.

The over simplistic attitude was modified when the function of the spindle intrafusal secondary endings and group II afferent fibers, revealed long ago (Matthews, 1972, 2006), was re-evaluated thanks to reports emphasizing their role in stance and locomotion, based on findings from normal subjects and neuropathic patients (Corna et al., 1995; Mazzaro et al., 2005). The group II fibers are about as numerous as the Ia fibers (Hunt, 1954) and, in spite of their conduction velocity being about half that of the former (about 30 wrt 60 m/s, in man), they play a paramount role in the afferent control of quiet and perturbed stance (Schieppati and Nardone, 1997, 1999; Nardone and Schieppati, 1998; Simonetta-Moreau et al., 1999; Bove et al., 2003) and of locomotion (Mazzaro et al., 2006).

Those findings have led to somewhat re-dimension the role of the spindle primary afferent fibers in the control of posture and gait. If their inflow is down-weighted during stance, in favor of their companion group II fibers, which are more appropriate for transducing slow changes in muscle length (Matthews, 1977) and have more positive reflex effects (e.g., production of a larger EMG burst in the postural muscles, diverging excitation to both legs’ motoneurons) (Corna et al., 1996; Schieppati and Nardone, 1997), what is left to the primary endings and Ia-afferent fibers to do?

There are excellent books chapters and review papers that place proprioception in the context of the global control of movement by the human nervous system (e.g., Prochazka et al., 2000; Pierrot-Deseilligny and Burke, 2005). Some address the interaction of proprioceptive information and its modulation with the operation of the spinal circuits (e.g., Windhorst, 2007). Others address the sensing of limb position and limb movement, originating in the spindles, emphasizing the existence of two separate senses, and point to the contribution of centrally generated motor command signals (e.g., Proske and Gandevia, 2009). In a more recent review paper, Proske and Gandevia (2012) expanded their target to include the senses of position and movement of our limbs and trunk, the sense of effort, the sense of force, and the sense of heaviness, and the effects of exercise and aging on proprioceptive sense. The present short review intends to summarize recent findings on the effects of activation of the Ia spindle afferent fibers, with specific reference to body orientation in space during stance and locomotion and to perception of motion in space. In particular, attention is focused on the neck proprioception and on its activation by muscle vibration or contraction, considering both immediate and long-term effects.

## Muscle Vibration is a Powerful Tool for Activating the Primary Endings of Muscle Spindles

In spite of the wealth of knowledge on proprioception and on the role of the primary afferent spindle fibers, novel information on less obvious but not less important roles, is adding up continuously, also thanks to the use of time honored experimental procedures, like lesion or stimulation. As to the former, mother nature helps by providing us with appropriate models featuring loss of large-diameter sensory fiber function (e.g., peripheral neuropathy, as briefly mentioned above). As to the latter, a rough though harmless and selective way of activating the primary spindle endings is muscle vibration (Bianconi and Van Der Meulen, 1963; Burke et al., 1976a,b; Roll et al., 1989a).

Vibration (~100 Hz) is a potent stimulus for the primary endings of the muscle spindle, less so for the secondary endings and the Golgi tendon organs (Roll et al., 1989a). For instance, vibration can induce a tonic contraction of the vibrated muscle (De Gail et al., 1966; Schieppati and Crenna, 1984), but, in addition to segmental responses, it also produces global effects. In standing subjects, leg muscle vibration elicits illusions of position that provoke postural reactions dependent on the new illusory postural set. Achilles’ tendon vibration while standing produces an inclination of the body backwards (Eklund, 1972; Thompson et al., 2007). This may be an automatic postural reaction to the “illusion” of forward displacement (or of triceps lengthening, as conveyed by the vibration-induced increased discharge of Ia fibers), since restrained subjects adjusted their body backward via a joystick when allowed to do so (Ceyte et al., 2006). These and other studies on this topic have led to the proposition that our sense of verticality may depend to a large extent on proprioception (Hlavacka et al., 1992; Barbieri et al., 2008; Barra and Pérennou, 2013).

## The Neck is the Functional Link between Head and Body

Proprioception of the neck, as also of the axial muscles, has a powerful body-orienting effect during quiet stance and locomotion. Such a peculiar influence must have evolved with the neck itself and with the need to counteract gravity, when our ancestors emerged from the water (Jouffroy, 1992). Fish have no neck, and the axial muscles have basically a medio-lateral action during swimming. For their orientation in space, the function of the lateral-line system is enough (Webb, 1989). With terrestrial life and erect bipedal posture and heavy, mobile head, the interaction of neck, and trunk proprioception with the vestibular sense has reached a highly developed grade. Since vestibular signals cannot distinguish whether the head or the whole body is moving when the head moves on a stationary trunk, the neck-proprioceptive input provides the necessary information about head movements relative to the trunk. Accordingly, neck muscles are richly endowed with spindles, which are highly sensitive to head yaw rotation (Chan et al., 1987). With the development of the neck muscles and their function (head yaw rotation, roll inclination, flexion, and extension), a unique mode of control has arisen, whereby rotation is produced by activation of the sternocleidomastoideus (SCM) muscle of one side (opposite to the direction of rotation) and of the dorsal neck (DN) muscle group of the same side as the rotation. For instance, during voluntary head turning to the left, right SCM, and left DN are agonists, as are both SCM during head flexion, and both DN muscles during the usual antigravity tonic action and voluntary head extension (Mazzini and Schieppati, 1992). Such control must rest on the concurrent operation of separate ipsi- and contralateral descending pathways (Zangemeister et al., 1982; Mastaglia et al., 1986; Beimborn and Morrissey, 1988; Gandevia and Applegate, 1988; Conley et al., 1995; Mayoux-Benhamou et al., 1997).

The functional relevance of the neck as a crucial segment in the head and trunk relationship is attested by the strength of the cervico-collic reflex and the vestibulo-collic reflexes. Activation of proprioceptors in the neck evokes cervico-collic reflex, which works in combination with vestibulo-collic reflex for the head stability and body posture. Signals interact downstream at the level of the spinal cord and upstream at the level of the vestibular and reticular nuclei (Pompeiano, 1979; Wilson and Peterson, 1988). Animal data are available on the operation of these reflexes in the pitch, yaw, and roll plane (Peterson et al., 1985; Dutia and Price, 1987; Zennou-Azogui et al., 1993) and on the effect of limb proprioception on these reflexes (Rosenberg et al., 1980). The two reflexes appear to behave approximately linearly, both individually and in combination (Peterson et al., 1985), whereby the dynamic of these reflexes and their spatial organization assure a correct response to prevent oscillation of the head on a stationary body. In human, Guitton et al. (1986) found that the contribution to a head stabilization task of the short-latency cervico-collic and vestibulo-collic reflexes may be unimportant, while longer-latency effects can be as powerful as vision. We found no data specifically concerning the effects of neck muscle vibration on cervico-collic and vestibulo-collic reflexes in man. Likely, these reflexes could modulate perception and orientation by way of their effects on head-in-space and head-on-trunk posture. In turn, these effects might be modulated by vibration. Research is needed to get insight in the interaction between reflexes and motion perception.

### Neck-proprioceptive influence on body orientation

Simple slow head turns can result in lateral displacements of the body’s center of mass toward to the “occipital” side, particularly so in the presence of a tonic level of spindle discharge from leg muscles (Gurfinkel et al., 1995). Continuous vibration of the DN muscles, bilaterally, produces a reactive response in the sagittal plane consisting in a *forward* inclination of the body (Lekhel et al., 1988; Kavounoudias et al., 1999; Ivanenko et al., 2000). Various explanations for this may be entertained. Robust spindle discharge normally ensues when gamma-MNs are active, as during a tonic voluntary neck extension. Illusion thereof would produce forward body inclination, in order to align the head with the vertical again. Other explanations are plausible. Ivanenko et al. (2000) suggested that, since the vestibular input is constant, the head may well be considered stationary in space and the neck flexed (as if the DN muscles were elongated) on the trunk inclined backwards. The subjects would react to the illusion of the body center of mass being displaced forward, and would be pressed to propel the body forward. Haptic supplementation offered by a touch on a firm surface (Bove et al., 2006b) and vision (Bove et al., 2009) modulate the effects of neck vibration on posture in amplitude and time, indicating a key role of multiple sensorimotor integration for body orientation in space. It could be argued that our nervous system weights the proprioceptive inflow according to its priorities, which may lead to “compensatory reactions” aimed at maintaining the task variable stationary (Lockhart and Ting, 2007).

Neck vibration also influences the perception of body position in the yaw plane, without necessarily producing postural changes in response to equilibrium challenge. Unilateral vibration influences the subjective straight-ahead (SSA) perception, inducing a disparity between subjective perception and objective position of the body midline, and determines an illusory movement of the head and of the visual target (Biguer et al., 1988; Roll et al., 1989b; Taylor and McCloskey, 1991; Karnath et al., 1993; Lekhel et al., 1997; Ceyte et al., 2006). The SSA (detected by asking the subject to point to a remembered visual target presented before the vibration or to point to their own nose) and the visual target representation are displaced during neck vibration (Taylor and McCloskey, 1991; Seizova-Cajic et al., 2006). The SSA moves toward the same side as the vibrated DN muscle, while the visual target moves toward the opposite side (Biguer et al., 1988). The shifts of SSA and visual target are in the opposite direction when the SCM is vibrated. This is consistent as DN and contralateral SCM act as a synergistic pair during head rotations. Therefore, illusory head movement correlates with the illusory target movement (Taylor and McCloskey, 1991) during neck muscle vibration. Conversely, eye movements and illusory perception do not correlate, since vibration induces little or no change in eye movements (Lackner and Levine, 1979; Seizova-Cajic et al., 2006). Neck muscle vibration seems to influence primarily the relative position of the body with respect to space creating an illusory head deviation. This illusory head deviation corresponds to the real head deviation induced by contraction of the vibrated muscles. Emphasis on the neck proprioception is dictated by the topic of the present review; however, we would note that, among other things, transcutaneous electrical nerve stimulation in the region of the neck (Pérennou et al., 2001), wedge-prism exposure (Rode et al., 2003), or podokinetic stimulation (Scott et al., 2011) can also affect the SSA, possibly through activation of brain functions related to multisensory integration that help restore the body proprioceptive representation.

### Neck proprioception and self-motion perception

Self-motion perception depends on the integration of sensory signals about body movement from vestibular, visual, proprioceptive, auditory, and kinesthetic signals. Can neck proprioception interfere with the conscious perception of self-motion? Several studies have examined the convergence and the interaction between neck and vestibular input at level of the vestibular nuclei (Anastasopoulos and Mergner, 1982; Manzoni, 1988), the cerebellum (Manzoni et al., 1998; Brooks and Cullen, 2009; Luan et al., 2013), and the parieto-insular vestibular cortex (Shinder and Newlands, 2014). In addition, the dynamic interactions of neck proprioceptive and vestibular inputs in the perception of body movement have been systematically described (Mergner et al., 1991, 1992, 1998; Mergner and Rosemeier, 1998), and a linear summation mechanism between the two signals has been proposed (Karnath, 1994; Mergner and Rosemeier, 1998; Bottini et al., 2001). While combinations of dynamic vestibular and neck proprioception activity have been widely analyzed, little information is available on the influence of tonic, prolonged proprioceptive signals on the self-motion perception of vestibular origin (Cullen, 2012, 2014; Medrea and Cullen, 2013).

#### Neck muscle vibration modulates self-motion perception of vestibular origin

The brain continuously keeps track of the body movement, in order to establish the instantaneous spatial relationship between self and the world. In man, motion perception can be estimated by having standing subjects oscillate in the yaw plane in the dark, and tracking with a pointer the remembered position of an earth-fixed visual target. Panichi et al. (2011) use a modified version of this protocol, whereby the cyclic left–right rotation was of equal amplitude but had asymmetric velocity. This stimulus causes a strongly biased perception of movement due to the vestibular dynamic properties (Panichi et al., 2011; Pettorossi et al., 2013a), since vestibular signals promptly indicate fast head movements, while they are poor at sensing very slow movements (Goldberg and Fernandez, 1971; Kolev et al., 1996; Massot et al., 1999; Valko et al., 2012; Tremblay et al., 2013). By continuing the asymmetric vestibular stimulation, the bias in motion perception progressively increased, whereby the gain of the tracking response gradually and continuously increased during the fast rotation cycle and decreased during the slow rotation cycle (Pettorossi et al., 2013a). This way of stimulating the vestibular system proved to be appropriate for showing the neck influence on self-motion perception, since symmetric whole-body rotation was not able to disclose sizeable and unambiguous effects of superimposed neck muscle vibration.

**Figure 1:**
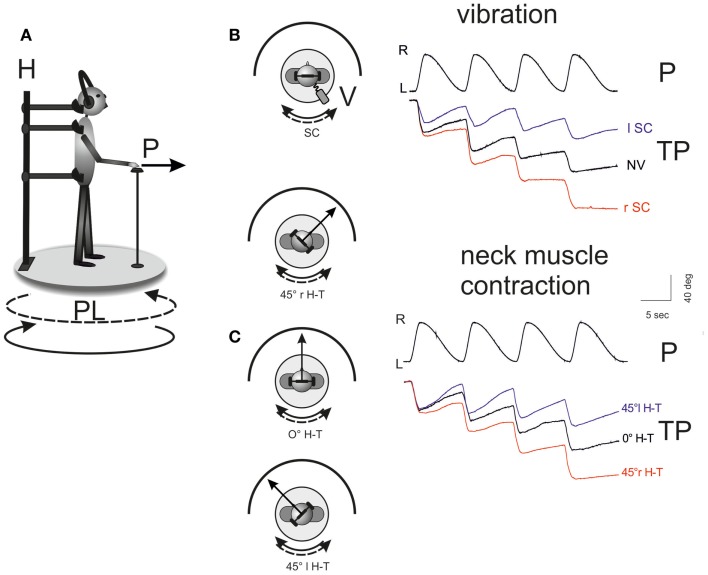
**Influence of unilateral neck muscle vibration on the self-motion perception elicited by whole-body rotation**. **(A)** Representation of the experimental setting. Subjects stood on a computer-controlled rotating platform in the dark (rotating platform, PL; pointer, P; holder, H). Circular lines indicate the fast platform rotation to the right (solid) and the slow rotation to left (dashed). Subjects were asked to manually track with the pointer (P) a remembered light spot (diameter 1 cm) presented before the test in front of them. Four consecutive asymmetric oscillation cycles were administered, made of two sinusoidal half cycles of equal amplitude but different frequency. **(B,C)** Tracking task recording during the vestibular stimulation (top, fast rotation to the right) and the effect of the conditioning maneuvers. The traces of platform oscillation (PL) and tracking position (TP) are shown during four cycles of asymmetric rotation. The remembered target position is progressively shifted toward the side of slow rotation (left) due to the properties of the vestibular system (NV, no vibration; 0°H-T, head in the primary position). Right Splenius Capitis (SC) muscle vibration **(B)** or maintaining the head rotated to right **(C)** induced an enhancement of the motion perception to the right during the asymmetric rotation, as shown by the displacement of the target representation. Conversely, left SC vibration and head active rotation to the left reduced motion perception and diminished the error in the target representation. In **(B)**, in the inset, is reported the schematic drawing of the platform rotation during 100 Hz frequency, 0.8 mm amplitude vibration (V: vibrator device) of right and left Splenius Capitis (rSc, lSC). In **(C)**, the effects of the three conditions of head-trunk positions (H-T angle) are reported: 0°, 45° to the right (r) and to the left (l) [adapted from Panichi et al. (2011)].

The motion perception bias produced by the asymmetric vestibular stimulation was strongly modified by unilateral neck muscle vibration or contraction or both (Panichi et al., 2011). These maneuvers doubled or annulled the bias, depending on the side of vibration or direction of head active deviation. Vibration of the DN or SCM muscle with the head in primary position differentially influenced the perceived rotation during asymmetric oscillation, coherently with their effect on head yaw voluntary rotation (Figure 1). The sign of the influence on the perceptive “bias” was opposite, while its amplitude was comparable. For instance, vibration of the left SCM produced an exaggerated perception of body rotation to the right (the sense of the fast cycle of whole-body rotation), while vibration of the left DN muscles almost canceled the bias in the vestibular-induced perception of rotation. Therefore, by enhancing the spindle firing from the muscles that turn the head, say, to the right, the sensitivity of the brain to whole-body rotation to the right was enhanced. Tonic active (but not passive) head deviation superimposed to the asymmetric whole-body oscillation also enhanced movement perception when the head was turned toward the side of the fast rotation and decreased it with opposite deviation (toward the site of the slow rotation) (Figure 1).

Therefore, the vestibular-evoked perception of body rotation is enhanced by neck-proprioceptive input as a function of the muscles’ action (turning the head, or trunk, to the right or to the left) rather than of their anatomical position (right or left of the midline). This effect may be useful for increasing the gain of the perception of motion in the presence of intense active rotation of the body, when the body movement must follow the direction in which the head turns, a condition that may require a superior perception for a better performance of the goal-directed movement (Panichi et al., 2011).

#### Does vibration mimic passive muscle lengthening or muscle contraction?

Vibration at the appropriate frequency (80–120 Hz) is as good a stimulus for the spindles of the neck muscles as it is for other body muscles. In spite of the uniqueness of the neck muscle spindles (Richmond and Abrahams, 1979; Price and Dutia, 1989), they are endowed with fusimotor fibers as are almost all body muscles. Therefore, the spindle discharge may well be larger during voluntary contraction (even more so for isometric than shortening contractions) than during passive lengthening of the muscles. Hence, the “illusion” of lengthening produced by vibration may as well conceal the illusion of muscle contraction. Accordingly, passive head deviation (without vibration) had no significant effects on the vestibular-evoked self-motion perception (Panichi et al., 2011). This implies that deliberate activation to keep the head deviated is necessary, while neck muscle lengthening induced by passive head rotation may be not sufficient. This must be a different process from that leading to gating of afferent signals to somatosensory cortex during active movement (Williams and Chapman, 2002; Barnett-Cowan and Harris, 2011). Based on others’ findings (e.g., Inglis et al., 1991), on Panichi et al. (2011), and Schieppati and Pettorossi (2014), one can deduce that vibration-induced and contraction-induced effects both depend on a strong discharge of the primary afferent spindle fibers.

Notably, however, the intensity of the perceptive effects produced by vibration and deliberate muscle contraction can differ due to the motor command (or its “efference copy”) reaching the same centers responsible for the perceptive responses [Panichi et al., 2011; see for a discussion Feldman et al. (2013)]. The efference copy, by definition, is ahead of the motor performance, and may not correspond to the desired motor effect. It could be argued, based on the ample equivalence of the effects of vibration and contraction on self-motion perception (Schieppati and Pettorossi, 2014) that perception is more driven by real movement than by the intention to move, if it has to have a functional meaning. As a corollary, since the secondary spindle endings are hardly activated by vibration but are certainly activated by the fusimotor discharge, the similarity of the effects of vibration, and contraction suggests that the secondary endings may be not relevant for eliciting the perceptive responses discussed here.

### Neck proprioception and body orientation during locomotion

Unilateral vibration of the neck muscles in normal subjects while stepping-in-place or walking produces, in the case of SCM, body turns to the side opposite to vibration, while in the case of DN muscles subjects deviate from the straight-ahead toward the same side as the vibrated muscles (Bove et al., 2001, 2002). The vibration-induced “orienting” effect is also common to other axial muscles, stimulated when the vibrators are in a paraspinal position at the toraco-lumbar junction (Schmid et al., 2005). Among the paraspinal muscles, the multifidus, rotatores, and semispinalis muscles rotate the vertebral column and the trunk to the opposite side [the erectors spinae also receive the vibratory stimulation when the vibrator is placed on the lumbar back, but their role would be that of a stabilizer rather than a rotator, see Kumar et al. (2002)]. Interestingly, axial muscles have a larger spindle density than other muscles (Voss, 1971; Banks, 2006). Mapping of several muscles within the same subjects during ground locomotion has confirmed the notion that only axial muscles (as opposed to limb muscles) are capable, when vibrated, of producing major, clear-cut deviations of the walking trajectories eyes closed (Courtine et al., 2007).

It is worth noting that not only neck or trunk muscle vibration but also galvanic vestibular stimulation induces major effects on the trajectory of the walking path (Fitzpatrick et al., 1999). Moreover, changing head posture changes the interpretation of the galvanic vestibular signal for balance and orientation responses (Fransson et al., 2000; Deshpande and Patla, 2005; Fitzpatrick et al., 2006). Thus, vibration-evoked responses from axial muscles might disclose interesting properties of vestibular influences on the control of body orientation. Clearly, the axial muscles are an important source of information about head and trunk orientation in space, and their discharge provides the CNS with cues about body orientation and rotation in space, which are then somehow transmitted to the centers controlling locomotion. Remarkably, though, subjects are not aware of any head or body yaw deviation during walking or rotation during stepping-in-place with vibration, and are always surprised by their unexpected position in space at the end of the trials, indicating that neck proprioception *per se* may not produce strong *conscious* perception of self-motion. In a similar manner people with vestibular dysfunction when asked to step in place with eyes closed are surprised by their change in body orientation.

The complexity of the underlying mechanisms can be appreciated by the fact that trajectory deviations by vibration are only obtained when locomotion is in progress. If the unilateral vibration starts before subjects initiate stepping, both feet on the ground, no obvious deviation is detected (Schmid et al., 2005). This seems to be in line with the notion that orientation in space is not only the result of an automatic sensory integration process but also depends on awareness of the orientation of the body segments, including the feet (Lyon and Day, 2005), very much as occurs for the sense of verticality (Barra and Pérennou, 2013).

Interestingly, in cervical dystonia, patients stepping-in-place show non-systematic body rotations during vibration of SCM. In addition, rotations are smaller than in normal subjects, and the confidence intervals in the patient population are about twice as much as those obtained for the normal subjects (Bove et al., 2004). It seems that in many patients the reference system used in the control of body orientation in space is either refractory to the lateralized proprioceptive neck input [also the effects on the standing body orientation are attenuated in cervical dystonia; see Lekhel et al. (1997) and Bove et al. (2007)], or modified such that the input from either sides produces small or even “wrong” effects. Note that, in a seated patients, long-lasting vibration of the dystonic muscle produced persistent reorientation of the head, as a sign of the function of segmental circuitry subserving head rotation (Karnath et al., 2000). Perhaps, this relative obliviousness of neck proprioception in the context of whole-body orientation in dystonia is connected to plasticity in the supraspinal circuits and centers integrating the neck input, shaped by the long-term asymmetric spindle inflow from one side of the neck (Münchau and Bronstein, 2001). Likewise, in Writer’s Cramp (Grünewald et al., 1997), the sensation of movement produced by the vibratory stimulus was not perceived normally in the dystonic patients, as if misinterpretation of Ia-afferent discharges also occurred (Wagner et al., 2008).

Body orientation during locomotion and stepping-in-place must be instant-by-instant coherent with the SSA. Thus, unilateral vibration of a neck muscle must exert an influence on the centers that produce the gait pattern, not unlikely that exerted by the galvanic stimulation (Fitzpatrick et al., 1999; Iles et al., 2007) or by the voluntary command for turning (Courtine and Schieppati, 2003). Notably, during volitional locomotion along a curved trajectory, head yaw anticipates body yaw (Courtine and Schieppati, 2003). The head turns more than dictated by the heading change, probably as a sign of anticipation: head orientation with respect to the body antecedes the body heading at the next step, and so on for the successive steps. This, however, may be not an obligatory coupling during volitional locomotion, since Cinelli and Warren (2012) argue that head rotations *per se* are neither necessary nor sufficient to induce changes in the direction of locomotion when walking to a goal.

The asymmetric vestibular stimulation mentioned in a preceding paragraph influences the SSA because the information associated with the fast rotation to one side largely prevails, while that associated with rotation to the opposite side (of equal amplitude, but slower) weakens the sense of the rotation (Pettorossi et al., 2013a,b). In turn, the unilateral vibration of neck muscle strongly influences the effect of the asymmetric whole-body rotation (Panichi et al., 2011). There must be some algebraic effect of the two stimulations at some central site (the bias in the self-motion perception of vestibular origin may be either enhanced or annulled depending on the vibrated neck muscle). If this is tenable, one would argue that the rotation during stepping-in-place or the deviation during locomotion induced by unilateral vibration of axial muscles depend on the priority of the moving body, i.e., continuously keeping the current SSA in front of it. Interestingly, blindfolded subjects have a tendency to walk in a large circle. Souman et al. (2009) suggested that veering from a straight course may result from accumulating noise in the sensorimotor system, without an external directional reference to recalibrate the subjective straight ahead. It is not unlikely that minor but enduring asymmetric proprioceptive input, not periodically checked by vision, may causes people to walk in circles as a result of errors in their SSA.

## Aftereffects of Neck Vibratory Stimulation

Aftereffect is by definition an aspect of adaptation due to the history of stimulation, which persists after the end of the stimulus (Helson, 1948). The aftereffect can be simply a continuation of the effect or it can show responses of opposite sign. Neck-proprioceptive stimulation, especially after prolonged vibration to the muscles, should elicit aftereffects. Other systems, apart from the proprioceptive, are also involved in postural control and space orientation and show aftereffects. The vestibular and the optokinetic systems, after prolonged stimulation, exhibit responses that are initially coherent with those induced by the stimulus (post-rotatory nystagmus, PRN; optokinetic after-nystagmus, OKAN) (Brandt et al., 1974; Waespe and Henn, 1978; Clement et al., 1981; Koenig and Dichgans, 1981; Lisberger et al., 1981; Maioli, 1988; Pettorossi et al., 1999). Shortly afterward, these responses reverse their sign, typically showing after-nystagmus of the opposite sign (PRN II and OKAN II). These responses may be due to habituation taking place in the central optokinetic and vestibular circuitry. Neck muscle proprioception activation can also produce effects on body orientation that outlast the vibration train. These persistent effects would not be produced by reflex adaptation or by proprioceptive receptor post-discharges, as if previously activated spindles continue firing (Ribot-Ciscar et al., 1996). They do not reverse in sign, and are possibly linked to a specific central-integration process.

### Aftereffect on balance

The inclination of the body induced by symmetric DN muscle vibration is in the *forward* direction both during and after the end of stimulation (Lund, 1980; Ivanenko et al., 1999, 2000; Kavounoudias et al., 1999; Bove et al., 2006a,b). The aftereffect on posture can last several minutes (Wierzbicka et al., 1998). As mentioned in Duclos et al. (2004), similar aftereffects were found not only after prolonged vibrations applied to neck muscles but also after prolonged voluntary contraction of the same muscles.

Conversely, a different aftereffect on balance displacement has been reported when vibration is applied to non-axial muscles. For instance, backwards body inclination and trunk extension (Thompson et al., 2007), observed during vibration of soleus (Capicíková et al., 2006), invert to forward inclination after the end of the stimulus, albeit it with a large variability of the responses. An opposite aftereffect has also been observed for joint movement perception (Seizova-Cajic et al., 2007). Habituation of the illusion of elbow extension occurs during biceps brachii vibration, and after vibration ends a flexion illusion subsides. It appears that the direction of the vibratory aftereffect is coherent with that observed during vibration when neck muscles are vibrated but has an opposite direction when limb muscles are vibrated.

### Aftereffect on the subjective straight-ahead

The vibration-induced deviation of the SSA (or the space in front of our nose) (Taylor and McCloskey, 1991; Seizova-Cajic et al., 2006) persists in the same direction as during the stimulus, when vibration stops. Depending on the duration of the stimulus, the aftereffect on SSA can last several hours or days in both normal subjects (Karnath et al., 2002) and neglect patients (Ferber and Karnath, 1999; Schindler et al., 2002; Johannsen et al., 2003).

On the other hand, the motion of an illusory visual target induced by vibration reverses its direction at the end of the vibration. Therefore, the illusory visual target movement, which is coherent with the SSA displacement during vibration, becomes incoherent when vibration is discontinued (Lackner and Levine, 1979; Biguer et al., 1988; Taylor and McCloskey, 1991). To explain this discrepancy, Seizova-Cajic and Sachtler (2007) have proposed that the aftereffect inversion of illusory target movement is primarily related to the presence of visual signal, since the inversion is absent when the target is not seen during vibration but only after vibration.

### Aftereffect on self-motion perception

**Figure 2:**
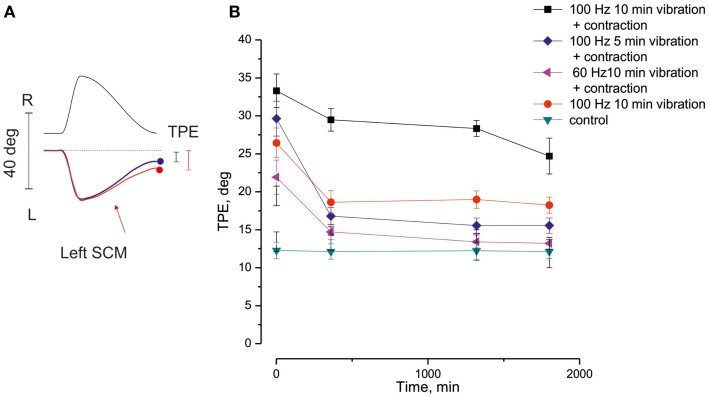
**Long-lasting aftereffect of neck muscle vibration on the self-motion perception**. **(A)** Traces of the tracking of remembered visual target during a single cycle of asymmetric rotation (0.15 Hz frequency, asymmetry 80%). Top: platform oscillation trace. Bottom: tracking traces, with (red) and without (blue) left sternocleidomastoideus (SCM) muscle vibration. Note the tracking position error (TPE) at the end of the rotation cycle. The position error between the real position of target and its representation (vertical bars), produced by the asymmetric whole-body rotation, is further increased by the left SCM vibration (red trace). **(B)** The time course of the enhancement of TPE after SCM vibration. In abscissa: time after vibration, at which the asymmetric oscillation cycle is administered; in ordinate: amplitude of TPE. Note that the TPE enhancement and its persistence is influenced by the duration and frequency of the vibration train and by the simultaneous contraction of SCM [adapted from Schieppati and Pettorossi (2014)].

The large modulation produced by neck muscle vibration in the movement perception of vestibular origin, mentioned above (see Section “Neck Muscle Vibration Modulates Self-Motion Perception of Vestibular Origin”), is present not only during the on-going vibratory stimulation but also after it (Schieppati and Pettorossi, 2014) (Figure 2). The enhancement of the vestibular-elicited motion perception bias (in the direction of the head deviation, or in the direction that the vibrated muscle would induce if contracted), or the reduction of the motion perception bias (with the head deviated in the opposite direction or when an antagonistic muscle was vibrated), both persist at the end of the vibratory stimulus. The aftereffect endures minutes or hours depending on the duration and frequency of vibration and on the status of the vibrated muscle (relaxed or contracted). In passing, persistent aftereffects of proprioceptive origin have been observed not only in motion perception but also in completely different experiments and in other muscle groups. For instance, prolonged vibration of limb muscles induces long-term cortical excitability change (Marconi et al., 2008), enhancement of leg muscle power, and improvement of body balance (Brunetti et al., 2006; Filippi et al., 2009).

Neck vibration aftereffects on self-motion perception could be explained by plastic events occurring in the vestibular networks responsible for motion perception when there is an intense proprioceptive activation, able to drive persistent membrane and genomic synaptic changes (Grassi and Pettorossi, 2001; Wolpaw and Tennissen, 2001; Lynch, 2004; Straka et al., 2005; Pettorossi et al., 2011). A remarkable consolidation of the aftereffect is obtained with frequencies of 80–100 Hz, while below this range the persistence of the aftereffect is scarce. The greater efficacy of the high-frequency entails a stronger activation of the primary spindle afferents onto the central network. Higher frequencies may be also more apt *per se* to induce synaptic plasticity, since high-frequency stimulation induces learning processes in other afferent systems, while low frequencies tend to reduce such effect [Lynch, 2004; Stanton and Sejnowski, 1989; Bliss and Collingridge, 1993; Nicoll and Malenka, 1995; Pettorossi et al., 2013b; Scarduzio et al., 2013; Beste and Dinse, 2013; Seitz and Dinse (2007)]. *In vitro and in vivo* experiments with different types of afferent fiber stimulation, tactile (Dinse et al., 2003, 2011; Ragert et al., 2008), visual (Beste et al., 2011; Beste and Dinse, 2013), acoustic (Amitay et al., 2006), and vestibular afferents (Grassi et al., 1996; Grassi and Pettorossi, 2001; Pettorossi et al., 2013b; Scarduzio et al., 2013), suggest that high-frequency afferent fiber stimulation leads to long-term potentiation (LTP, Lynch, 2004) in several regions of the CNS, while low frequency to long-term depression or cancelation of previously induced LTP.

#### Muscle status

The status of the muscle during the vibration is critical for inducing the long-term aftereffect on self-motion perception. Tonic, isometric muscle contraction can increase both the amplitude and the duration of the perceptive aftereffect induced by vibration (Schieppati and Pettorossi, 2014). The enhancement of the aftereffect obtained by concomitant vibration and muscle contraction on self-motion perception is unexpectedly greater than that estimated by adding the aftereffects of both muscle contraction and vibration (Figure 2). Post-vibratory effects and post-contraction response show remarkable similarities, as studied in the arm muscles (Gilhodes et al., 1992). In a study based on a different paradigm and addressing the effects of a limb movement on movement orientation, repetitive active arm movements *against a load* induced lasting changes in the space representation when active movement repetition lasted for at least 10 min (Ostry et al., 2010). While isometric contraction increases the activation of muscle spindles in response to the vibration either by enhancing spindle sensitiveness through γ-motoneuron activity, or by facilitating a better diffusion of the vibration within the muscle thanks to its increased muscle stiffness (Burke et al., 1976b), muscle contraction (and its efference copy) superimposed to the vibratory stimulation would favor the build-up and consolidation of the influences on motion perception (Rymer and D’Almeida, 1980; Smith et al., 2009; Luu et al., 2011). In particular, the voluntary activation in concomitance with peripheral proprioceptive stimulation may lead to potentiation of the synaptic responses, where the peripheral input and central drive converge along the perceptive central pathway.

### Aftereffect on locomotion

The walking speed increment induced by bilateral vibration of the DN muscles, likely the consequence of the postural illusion mentioned above (Ivanenko et al., 2000), promptly subsides at the end of the vibratory train. Aftereffects on stepping induced by bilateral contraction of DN muscles, performed during stance, are also inconsistent, unless contraction has produced fatigue (Schmid and Schieppati, 2005), under which circumstance the stepping body tends to move backwards. On the other hand, unilateral neck muscle vibration shows non-systematic aftereffects on stepping direction. The body initially rotates toward one side (most often the same side as during the vibration administered during stepping) and rotate afterward toward the opposite side (Bove et al., 2002). Further, when vibration (or contraction, see above) is applied during stance, and stepping follows at the end of the vibration, the poor consistency of the aftereffect on body rotation is likely due on the details of the experimental procedure. Having both feet on the ground during vibration provides a fixed reference that attenuates the effects of the unilateral proprioceptive activation under this circumstance, much as light-touch does on the standing body orientation during vibration (Bove et al., 2006a,b).

**Figure 3:**
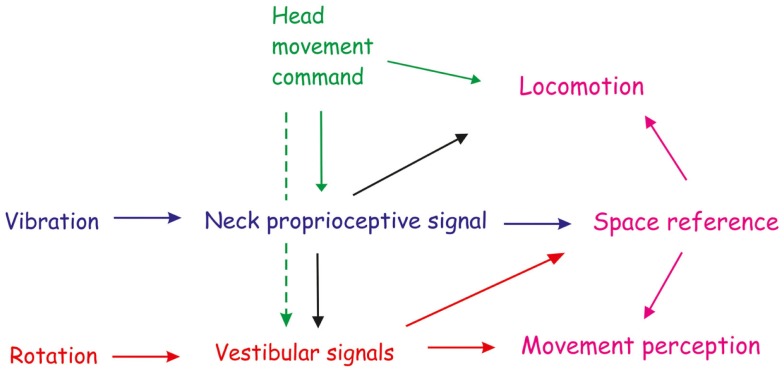
**Outline of the immediate and sustained interaction between neck proprioceptive and vestibular inflow, and of the motor command for shaping the space reference and influencing motor perception and locomotion**. Neck proprioception (blue arrow) and vestibular (red arrow) signals can modify movement and space perception separately. However, these changes can also result from the interaction between neck proprioception and vestibular system. Unilateral neck muscle vibration, mimicking either neck muscle elongation or contraction by γ-motoneuron drive, increases the vestibular responsiveness to yaw rotation (black arrow) in the direction corresponding to the head movement that would be produced by the contraction of vibrated muscle, and decrease when the antagonist muscle is being vibrated. Thus, tonic proprioceptive signals elicited by vibration can interact with the dynamic vestibular input and change the perception of whole-body rotation in yaw plane. Space reference and locomotion may be subsequently modified through this interaction (violet arrow). Therefore, neck proprioception directly, or indirectly by vestibular system, contributes to shape the subjective straight-ahead, the direction of walking and the self-motion perception. Prolonged neck muscle vibration also induces aftereffects in the straight ahead and motion perception in the same direction of the immediate effects. The persistence of the aftereffects depends on the intensity and duration of the vibratory stimulation. Descending signals for the head movement may also influence movement and motion perception (green arrow). They can contribute either to the effect and aftereffect by enhancing the peripheral signals from neck muscle (continuous line) or by directly changing the vestibular responsiveness to rotation (dashed line).

The information from the foot and leg status must interact with the supraspinal spatial orientation areas that influence spinal-level circuits for locomotion (Figure 3). Not unlikely, this is what occurs as a consequence of the so-called podokinetic adaptation, a whole-body yaw rotation during stepping-in-place eyes closed occurring after a period of stepping on a rotating treadmill (Weber et al., 1998). Interestingly, when asked to indicate their SSA with a laser pointer, these subjects demonstrated a significant shift in SSA regardless of whether they were standing or sitting (Scott et al., 2011). This would be in keeping with the notion that prolonged adaptive rotation of the feet may influence the SSA, and with the proposal that subjects track their SSA during the involuntary rotation aftereffect as much as they do with unilateral neck muscle vibration.

On the other hand, also the post-contraction facilitatory effect (Kohnstamm phenomenon), induced by prolonged and forceful deliberate body torsion, can modify the direction of an intended straight-ahead walking task, such that subjects walk along a curved trajectory in the direction of the preceding torsion (Ivanenko et al., 2006). This further supports the view that the proprioceptive inflow responsible for the orientation effects and aftereffect can be elicited by both vibration and contraction of axial muscles.

### The direction of the aftereffect

The aftereffect of neck muscle vibration seems to have the same direction as the effect observed during vibration: this would be true for the SSA displacement, standing balance displacement, and self-motion perception. On the other hand, the direction of the aftereffect would be opposite in the case of vibration of limb muscles. The reason for this divergence between neck and limb muscle vibration is not obvious. Neck vibration seems to consolidate the effect elicited during vibration, so that the effect is maintained even after the end of the stimulus. With limb proprioceptive vibration, on the other hand, the aftereffect of opposite sign could be attributed to the sustained stimulation leading to a habituation of the responses.

The consolidation of proprioceptive effects after neck muscle vibration such as those mentioned above, as opposed to habituation, would be explained by the different roles played by the proprioceptive system in the neck and limb muscles. It has been suggested that the tendency to habituation in the effect of limb proprioceptive activation is aimed to minimize the response to common environmental stimuli and increase the sensitivity to change (Seizova-Cajic et al., 2007), similarly to what happens in the vestibular and optokinetic system (Brandt et al., 1974; Waespe and Henn, 1978; Clement et al., 1981; Koenig and Dichgans, 1981; Lisberger et al., 1981; Maioli, 1988; Pettorossi et al., 1999). Conversely, the persistence of the effects on orientation and self-motion perception observed upon prolonged neck muscle activation supports the idea that the repeated proprioceptive information can shape a new reference frame for head and body around a new postural set (Karnath et al., 2002; Schieppati and Pettorossi, 2014). This might not be dissimilar from what occurs during motor learning (Lalazar and Vaadia, 2008). It is not unlikely that anomalous persistence of neck effects on motion perception may occur also as a consequence of pathological conditions presumably associated with persistent abnormal spindle discharge.

## Neck Muscle Spindle Primary Afferent Fibers Produce Immediate and Long-Term Influences on the Cognitive Body Representation

All in all, the behavioral and neurophysiological data reported above emphasize that proprioception from neck muscles contributes to the construction of cognitive representation of the body that includes position of limb segments, their hierarchical arrangement, and configuration of the segments in space. This does not normally enter into awareness, and may be primarily used for spatial organization of action (Haggard and Wolpert, 2005). Apparently, the proprioceptive information is processed according to the task performed, the time-interval during which the afferent volley takes place, the body segment from which the sensory inflow arises (neck and trunk), and concurrent stabilizing information. The information conveyed by the Ia fibers is rapidly transmitted to diverse parts of the CNS, and updates the brain on muscle length changes, and thus on movement. Its integration may occur at various level of the central nervous system, known to supervise the formation of reference frames for movement. Most likely, the vestibular nuclei, which receive neck muscle input, are the first stage for the integration of neck muscle vibratory signals and play a crucial role in conscious awareness of motion, spatial orientation, and navigation (Lopez, 2013) (Figure 3). The fastigial nucleus of the cerebellum is a site, in which computation of body motion is performed (Brooks and Cullen, 2009). Other anatomical substrates involved in the processing of neck muscle inflow are the motor cortex (Naito, 2004) and the parieto-temporal junction (Bottini et al., 2001). Interestingly, studies based on structural brain imaging [reviewed in Karnath and Rorden (2011), Blanke (2012), and Pfeiffer et al. (2014)] suggest that diverse subcortical (Clark and Taube, 2012) and cortical areas spanning the Sylvian fissure can be a substrate of the integration concerned in cross-modal interactions between somatosensory and vestibular signals (Bottini et al., 2013). These areas can be lesioned in various forms of neglect (Vuilleumier, 2013).

The effects of intense neck muscle vibration on self-motion perception during whole-body rotation are not restricted to the epoch of the stimulation but persist for an extended period of time. Vibration can lead to a consolidation of new space coordinate system by persistently modifying the self-motion perception of vestibular origin and interacting with the adaptive processes of the vestibular system (St George et al., 2011). This may be useful for adapting the perceptive (and consequently motor) responses to a novel postural set or motor bias, when proprioceptive activation persists for a sufficient period of time, thereby influencing the spatial references, motion perception, and locomotor orientation.

Admittedly, while vibration is an adequate stimulus for the rapidly adapting primary spindle terminals, vibratory trains are quite an unusual stimulation for the proprioceptive system, not least because it can signify anatomically impossible kinematics (Lackner and Taublieb, 1984; Seizova-Cajic et al., 2007). However, the effect of vibration mimics, at least in part, the effect of the γ-motoneuron activation, thereby functionally engaging the same pathway traveled during voluntary movement, and has been shown to have positive effect in various patients. It is on these premises that therapeutic effects of focal vibration may have a role in the armamentarium of the restorative neurology [see for a recent review Murillo et al. (2014)]. Long-duration trains of Ia firing, as induced by vibration, may disclose the capacity of proprioception to produce adaptive effects in an as yet unnoticed way. Recent findings by Yu et al. (2013) have shown in the cat that repeated exposure to cross-modal stimulation enhances neuronal sensitivity to the stimuli in the exposure set [see for a review Rowland and Stein (2014)]. By looking for aftereffects, Wright (2014) asked whether postural responses seen during discordant virtual-reality and physical vection stimulation involved adaptation, and described an aftereffect in the center of foot pressure, that could even last for a few days. New experiments dedicated to the observation of the effects of persistent activation of proprioceptors could provide novel insight into the plastic changes of our motor processes.

## Conclusion

Neck muscle inflow has prominent immediate and late effects on perception of body orientation and motion. Prolonged, intense proprioceptive input from neck muscles can induce persistent influences on self-motion perception and cognitive body representation (Figure 3). These plastic changes might adapt motion sensitiveness to lasting or permanent head positional or motor changes, like those accompanying movement disorders (see above) or those accompanying weightlessness (Roll et al., 1998). New experimental protocols based on these findings could open new avenues in the investigation of the consolidation of motor learning.

## Conflict of Interest Statement

No party having a direct interest in the results of the research supporting this article has or will confer a benefit on the authors or on any organization with which the authors are associated.
